# Does Systemic Chemotherapy Influence Skeletal Growth of Young Osteosarcoma Patients as a Treatment-Related Late Adverse Effect?

**DOI:** 10.3390/curroncol29060325

**Published:** 2022-06-04

**Authors:** Manabu Hoshi, Naoto Oebisu, Tadashi Iwai, Yoshitaka Ban, Hiroaki Nakamura

**Affiliations:** Department of Orthopedic Surgery, Osaka Metropolitan University Graduate School of Medicine, 1-4-3 Asahi-Machi, Abeno-Ku, Osaka 545-8585, Japan; evis@med.osaka-cu.ac.jp (N.O.); qq329xpd@opal.ocn.ne.jp (T.I.); ychbanchan@gmail.com (Y.B.); hnakamura@med.osaka-cu.ac.jp (H.N.)

**Keywords:** chemotherapy, osteosarcoma, height, skeletal growth, treatment-related late adverse effect

## Abstract

The aim of this study was to investigate the influence of systemic chemotherapy on the skeletal growth of young osteosarcoma patients as a treatment-related late adverse effect. We reviewed the height data of 20 osteosarcoma patients (13 males and 7 females) aged ≤18 years. The average (±SD) age at diagnosis was 14.5 (±3.3) years. The average follow-up interval was 89.6 months. After wide resection of the affected bones, reconstruction with tumor prostheses and auto-bone grafting was carried out in 11 and 9 cases, respectively. Pearson’s correlation coefficient was calculated to evaluate the association between actual and predicted (using Paley’s multiplier method) heights. *Z*-scores were used to compare the initial and final heights with the Japanese national growth curve. Actual and predicted heights were correlated according to Pearson’s correlation coefficient (*R* = 0.503). *Z*-analysis showed that statistical significance (*p* = 0.04) was noted for the height data *Z*-scores of patients between ≤10 years and >10 years at the final follow-up. Systemic chemotherapy did not reduce skeletal growth in young osteosarcoma patients as a late adverse effect based on two different evaluation methods. However, patients aged ≤10 years at diagnosis may develop a short stature after systemic chemotherapy.

## 1. Introduction

Osteosarcoma is the most common primary malignant bone tumor in adolescents and young adults. The standard treatment for osteosarcoma is neoadjuvant systemic chemotherapy and limb-salvage surgery with wide resection [1,2]. Recent multidisciplinary therapies have remarkably improved the prognoses of osteosarcoma patients, reporting a 5-year survival rate of over 65% [3,4]. A study on survival rate improvement also reported that the treatments for young patients receiving systemic chemotherapy have generated various kinds of late adverse effects [5]. Therefore, the interest in treatment-related late adverse effects among young cancer patients has increased.

Osteosarcoma most commonly affects patients between 10 and 19 years of age [6], which is also the period of growth spurts in adolescents. Therefore, we speculate that systemic chemotherapy during this active development period may reduce skeletal growth as a treatment-related late adverse effect. Short stature after cancer treatment is relatively common as a treatment-related late adverse effect in pediatric cancer patients, especially in pediatric leukemia and brain cancer [7,8].

However, studies concerning skeletal growth after osteosarcoma treatment are few. Therefore, we investigated the influence of systemic chemotherapy on skeletal growth among osteosarcoma patients.

## 2. Materials and Methods

This was a retrospective study. Between September 1985 and December 2019, a total of 48 patients aged ≤18 years and diagnosed with high-grade osteosarcoma were treated at our hospital. The inclusion criteria for this study were diagnosis with high-grade osteosarcoma at the age of ≤18 years, systemic chemotherapy, and availability of clinical data on patient height during initial diagnosis and final follow-up. During the final follow-up, patient age was >18 years.

Clinical information, including sex, age at diagnosis, affected site, height data at diagnosis and final follow-up, chemotherapy protocol, and surgical procedure, were examined.

The height was routinely recorded without shoes within one week after definite diagnosis. At the final follow-up, the height was measured based on the standing position on the healthy side. If necessary, the soles of the feet were heightened and adjusted to compensate for the shortening of the affected lower limbs. Height was measured after confirming that the heights of the pelvis on both sides were parallel to the ground. The height data of 17 patients was collected during routine follow-up in the outpatient department of our hospital or in another hospital. We performed telephone surveys with three patients and asked them to correct their height as much as possible.

To determine whether the height of young osteosarcoma patients treated with systemic chemotherapy grew as expected, the final height was compared with values predicted using Paley’s multiplier method [9]. These values were predicted according to a specific age and sex and were calculated using the formula M = Hm/H, where M is the sex and age-specific multiplier, Hm is the predicted height value at skeletal maturity, and H is the height at osteosarcoma diagnosis. At the time of final follow-up, the height of patients aged 18 years or older was substituted for the height value at the age of 18 years.

All patients included in this study are Japanese aged ≤18 years. Therefore, we used a registry based on Japanese data. The *Z*-scores of the height data at diagnosis and at final follow-up were determined based on Japanese national growth curve data [10]. Each *Z*-score was adjusted for age and sex.

The *Z*-scores at diagnosis and at final follow-up were compared among all patients, as well as between each sex and age group (≤10 years and >10 years).

This study protocol was approved by the Institutional Ethics Review Board of our hospital.

### Statistical Analyses

Pearson’s correlation coefficient (*R*) was calculated to compare the actual and predicted heights. *Z*-analyses were used for available data from the Japanese national growth curve, and the *Z*-scores were expressed as mean (± SD). These *Z*-scores have a normal distribution (mean of 0 and standard deviation of 1). The Mann–Whitney *U* test was performed for statistical comparison of the two groups. Statistical analyses were performed using Excel statistics software (version 2020; Social Survey Research Information Co., Ltd., Tokyo, Japan) for Windows, and *p* values of <0.05 were considered statistically significant.

## 3. Results

Patients were excluded if they presented with metastasis at diagnosis (*n* = 11), were managed with additional chemotherapy for newly appearing metastatic lesion after conventional chemotherapy during the follow-up (*n* = 7), or had amputation surgery (*n* = 2). Seven patients were excluded because of incomplete clinical data. A total of 28 patients were excluded. One patient was followed up with for less than one year.

In total, 20 patients (13 males and 7 females) fulfilled the inclusion criteria and were enrolled in the study. The average age (±SD) at diagnosis was 14.5 (±3.3) years (range: 9–17 years). The average interval from the diagnosis to the last follow-up was 89.6 months (range 13–325 months).

The tumors were located in the femur in 13 patients (proximal, 1; mid-shaft, 2; and distal; 10), tibia in 5 (proximal, 4 and distal, 1), and humerus in two (proximal, 2).

The treatment protocol for patients included in this study consisted of preoperative chemotherapy, wide resection, and postoperative chemotherapy. After wide resection of the affected bones, reconstruction with tumor prostheses and auto-bone grafting had been carried out in 11 and 9 cases, respectively.

Chemotherapy was based on the Osaka University Osteosarcoma (OOS)-D protocol [11] and Kanazawa (K)—2 protocols [12] in 18 and 2 cases, respectively. Both were modified with T12 protocols [1], and the treatments were composed mainly of doxorubicin, cisplatin, ifosfamide, methotrexate, and etoposide (Table 1).

**Figure 1:**
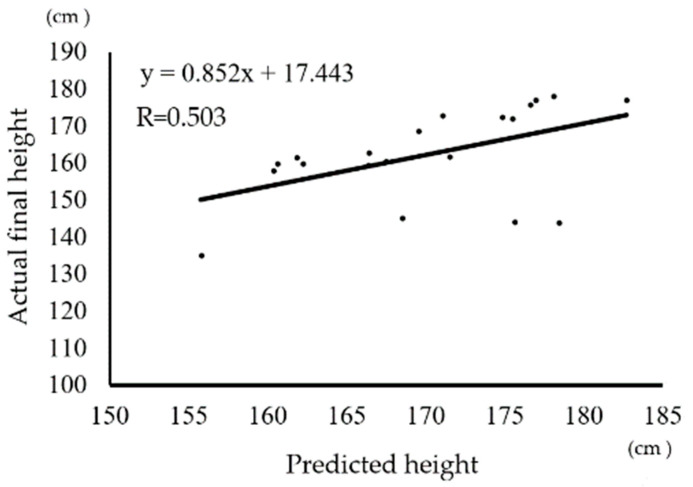
Relationship between actual and predicted height.

Figure 1 shows the relationship between the actual and predicted heights among the 20 patients with osteosarcoma. R, which was calculated to determine the accuracy of heights predicted using Paley’s multiplier method, is equal to 0.503. This means that the predicted height is correlated with the actual height.

There was a statistical correlation between the actual and predicted (using Paley’s multiplier method) heights, according to the calculated Pearson’s correlation coefficient (R = 0.503).

**Figure 2:**
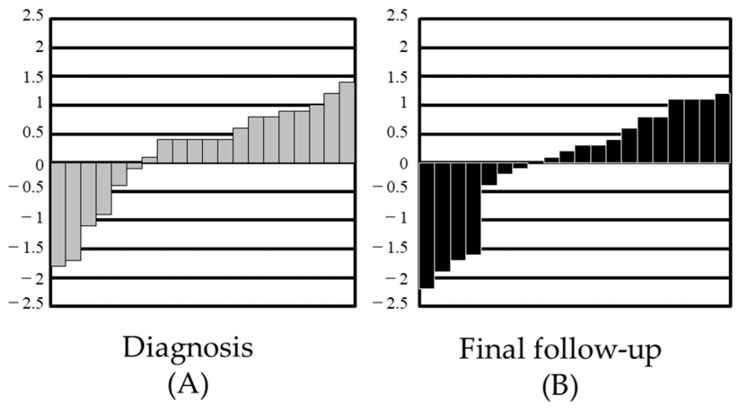
Distribution of standard deviations in height data: (**A**) standard deviation (SD) score at diagnosis; (**B**) SD score at final follow-up.

The Z-scores were calculated from the Japanese national growth curve, and the scores were arranged from low to high (Figure 2). The scores at the time of diagnosis (Figure 2A) were compared with those at the time of final follow-up (Figure 2B). Decreasing Z-scores could be seen among the final follow-up height data, but no statistically significant difference was observed.

**Figure 3:**
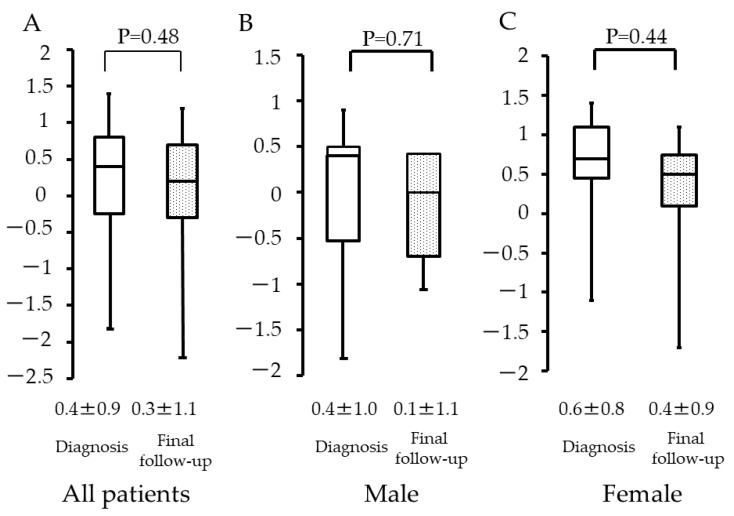
Height at diagnosis and final follow-up with respect to patient sex. (**A**) All patients. (**B**) Male patients. (**C**) Female patients.

Among all patients, the Z-scores were 0.4 (±0.9) and 0.3 (±1.1) at diagnosis of osteosarcoma and at the final follow-up, respectively (Figure 3A). Among male patients, the values were 0.4 (±1.0) and 0.1 (±1.1) (Figure 3B), and, among female patients, the values were 0.6 (±0.8) and 0.4 (±0.9) (Figure 3C), respectively. There was no statistical significance observed among these three groups (*p* = 0.48, *p* = 0.71, and *p* = 0.44).

**Figure 4:**
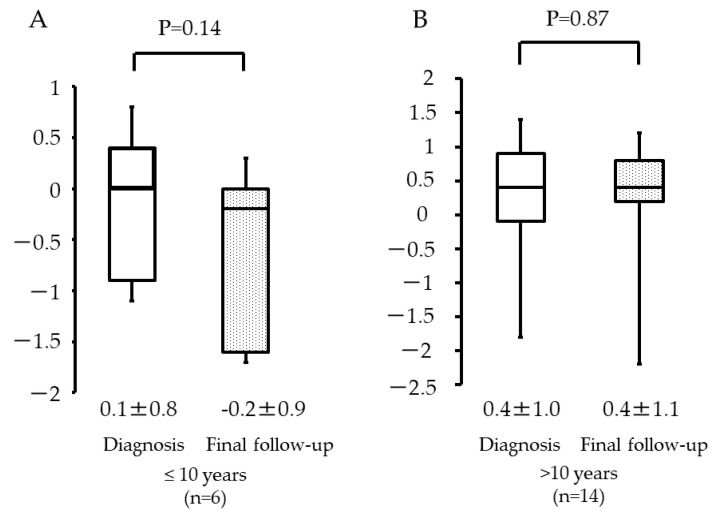
Height at diagnosis and final follow-up with respect to patient age: (**A**) ≤10 years; (**B**) >10 years.

Among patients aged ≤10 years, the Z-scores were 0.1 (±0.8) and −0.2 (±0.9) at diagnosis and at the final follow-up, respectively (Figure 4A). Among patients >10 years, the values were 0.4 (±1.0) and 0.4 (±1.1), respectively (Figure 4B). There was no statistical significance observed among these two groups (*p* = 0.14 and *p* = 0.87).

**Figure 5:**
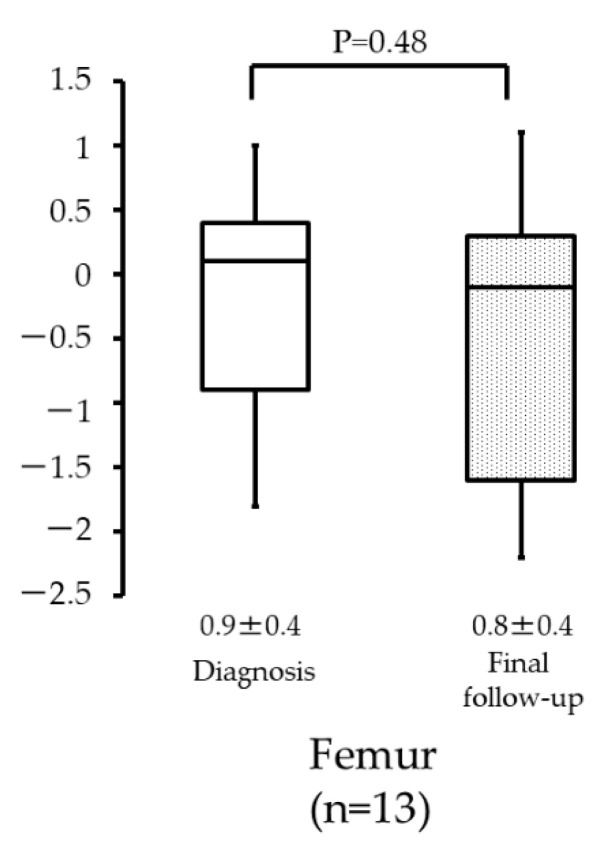
Height of patients with femur osteosarcoma at diagnosis and final follow-up.

Among all patients with femur osteosarcoma, the Z-scores were 0.9 (±0.4) and 0.8 (±0.4) at diagnosis and at the final follow-up, respectively (Figure 5). There was no statistical significance observed between these two groups (*p* = 0.48).

**Figure 6:**
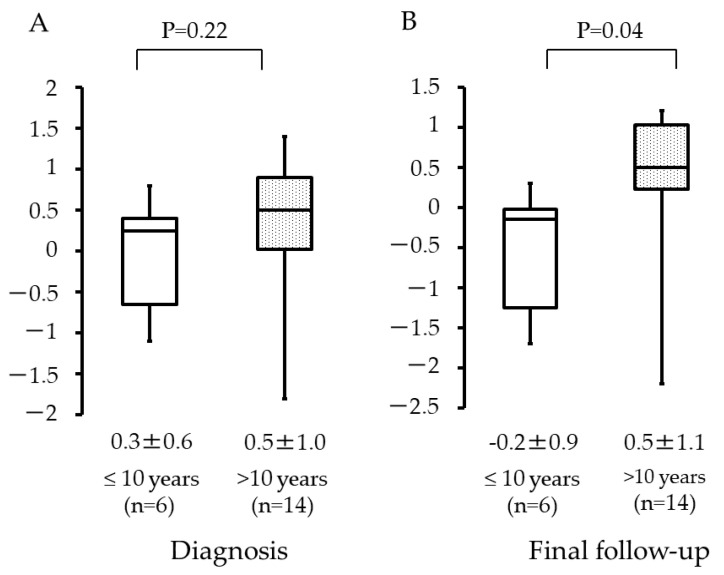
Height per patient age group, with respect to time: (**A**) Z-score of height at diagnosis for patients aged ≤10 years and >10 years; (**B**) Z-score of height at final follow-up for patients aged ≤10 years and >10 years.

The Z-scores at diagnosis were 0.3 (±0.6) and 0.5 (±1.0) for patients aged ≤10 years and >10 years, respectively (Figure 6A). The values at the final follow-up were −0.2 (±0.9) and 0.5 (±1.1), respectively (Figure 6B). There was no statistical significance observed at diagnosis (*p* = 0.22), but a statistical difference was observed at the final follow-up (*p* = 0.04).

## 4. Discussion

This study examined the influence of systemic chemotherapy on the skeletal growth of osteosarcoma patients aged ≤18 years at the time of diagnosis. We speculated that reduced height could be expected as a treatment-related adverse effect, since these young osteosarcoma patients underwent systemic chemotherapy during the active skeletal growth period. Based on our results, the height predicted by Paley’s multiplier method was correlated with the actual height at the final follow-up, which means that the actual final height was relatively within the prediction. Additionally, the *Z*-analysis using data from the Japanese national growth curve showed no significant difference in the height data at diagnosis and at the final follow-up. This study failed to prove that systemic chemotherapy significantly reduced the skeletal growth of osteosarcoma patients as a treatment-related late adverse effect.

Short stature in pediatric leukemia and pediatric brain tumor patients after cancer treatment is among the common treatment-related adverse effects, especially when radiation therapy had been applied to the brain and spinal cord [13,14]. The treatment-related adverse effects of systemic chemotherapy on the skeletal maturation of patients with malignant primary bone tumors have been previously investigated (Table 2). Glasser et al. demonstrated that patients with osteosarcoma and Ewing’s sarcoma who were treated with systemic chemotherapy did have height impairments when compared to the normal population [15]. Cool et al. also reported that 72 osteosarcoma patients had no significant difference in height at their last follow-up after systemic chemotherapy when compared to healthy individuals [16]. In contrast, Glig et al. showed that patients with osteosarcoma and Ewing’s sarcoma had shorter statures compared to predicted height values after systemic chemotherapy [17].

The prediction of height at skeletal maturity has been advocated by Bayley and Pinneau [18], Tanner et al. [19], and Roche et al. [20]. These previously reported prediction methods require complex data comprising radiographs measuring skeletal age, nude height, occurrence of menarche, and mid-parent height. In contrast, Paley’s multiplier method [9] is simple and purely based on sex and chronological age data. In our study, we compared predicted and actual height values. *R* was calculated to be 0.503, which means that the height predicted by Paley’s multiplier method is correlated with the actual height at the final follow-up. This result supports the idea that the final height of young osteosarcoma patients is not affected by systemic chemotherapy.

Paley’s multiplier method was based on height data from the National Center for Health Statistics. The population data were composed of healthy and multiethnic children [9]. However, our study involved Japanese patients; hence, we also needed to compare our data with the Japanese registry. The Japanese national growth curve was designed and is available for the evaluation of short stature among patients of various pediatric diseases. According to our results, there were no significant differences among the factors of sex and age for height data at the time of diagnosis and the final follow-up. These findings illustrate that skeletal growth in osteosarcoma patients was not affected by systemic chemotherapy.

A statistical significance was observed among the *Z*-scores of patients at the final follow-up (−0.2 (±0.9) and 0.5 (±1.1) for patients aged ≤10 years and >10 years, respectively). This result suggests that ages ≤10 years seem to be an important factor in predicting future short statue, when compared to patients aged >10 years who received the same systemic chemotherapy regimen. Previous studies have identified some factors as significant for predicting the risk of short stature after cancer chemotherapy, such as sex and age. Vinnna [7] and Sklar [21] reported that systemic chemotherapy for leukemia significantly reduced height especially in female patients. Moreover, height increase after treatment for leukemia has been suppressed in younger patients. Daiton et al. [13] reported that patients between 5 and 8 years had short statures after systemic chemotherapy. Similarly, Vilela et al. [14] reported that treatment for acute lymphocytic leukemia induced short stature in patients younger than 4 years.

The femur, which is the longest bone in humans, is also the most common location of osteosarcoma. Therefore, we speculate that the affected femur has a significant impact on the final height of osteosarcoma patients because skeletal growth may be interrupted by chemotherapy; however, no significant difference was observed in the height values at the time of diagnosis and the final follow-up.

Chemotherapy-induced short stature is thought to be the result of direct and indirect mechanisms. The direct effect may be growth plate damage caused by anticancer drugs [22,23,24], whereas indirectly, anticancer drugs may disturb the function of the hypothalamus–pituitary gland, resulting in growth hormone reduction [25]. During the follow-up, there were no patients with disturbed brain functions, including mental health. The standard systemic chemotherapy treatment for osteosarcoma patients is mainly composed of methotrexate, doxorubicin, cisplatin, and ifosfamide [1]. Among these key drugs, Leeuwen [22,23,24] reported that doxorubicin and methotrexate directly impaired increase in bone length in an in vivo animal model. Doxorubicin was reported to reduce plate height growth, affecting the proliferation zone, whereas methotrexate increased the height of the hypertrophic layer in the growth plate and disturbed the trabecular structure.

Our study has some limitations. This study was retrospective, and the small sample was comprised of patients of the same ethnicity (Japanese) from a single Japanese institution. Therefore, the results may not always be applicable to general osteosarcoma patients. The height was measured with a different type of instrument at the diagnosis and at the final follow-up; therefore, some errors were possible. The cumulative dose of the anticancer agents was not same, and the enrolled patients were being treated using two different protocols. Moreover, actual height values were compared with values predicted using Paley’s multiplier method but not with values that could be predicted using other methods, such as those by Bayley [18] and Tanner [19]. The use of other prediction methods was not possible because of a lack of radiographs of skeletal age, nude height, occurrence of menarche, and mid-parent height at the time of osteosarcoma diagnosis, which are data we do not routinely collect. Therefore, our evaluation of predicted height was limited to chronological age instead of skeletal age. The average interval from the diagnosis to last follow-up was 89.6 months, and a longer follow-up of over 10 or 20 years might find different results.

## 5. Conclusions

Orthopedic oncologists should be convinced and be able to inform pediatric osteosarcoma patients that systemic chemotherapy does not affect skeletal growth as a treatment-related late adverse effect, unlike leukemia and brain tumors.

## Figures and Tables

**Table 1:** Chemotherapeutic protocol for osteosarcoma patients in this study.

Author	Year	Pre-Operative Chemotherapy	Post-Operative Chemotherapy
Kudawara, et al. [11]	2013	DOX 80–90 mg/m^2^ + CDDP 120 mg/m^2^: 2 courses IFM 15 g/m^2^: 2 courses	MTX 10–12 g/m^2^: 4 courses
			DOX 80–90 mg/m^2^ + CDDP 120 mg/m^2^: 2 courses
			IFM 15 g/m^2^: 2 courses
Tsuchiya, et al. [12]	1999	DOX 60 mg/m^2^ + CDDP 100–120mg/m^2^: 3 courses	MTX 10–12 g/m^2^: 3 courses
		IFM 9g/m^2^ + ETP 180 mg/m^2^: 2 courses	DOX 60 mg/m^2^ + CDDP 100–120 mg/m^2^: 3 courses

DOX: Doxorubicin. CDDP: Cisplatin. MTX: Methtrexate. IFM: Ifosfamide. ETP: Etoposide.

**Table 2:** Previous studies concerning the skeletal maturity of patients with primary malignant bone tumours after cancer treatment.

Author	Year	Diagnosis	*N*	Reference	Outcome
Glasser, et al. [15]	1990	Osteosarcoma	68	United Kingdim cross sectional reference data	The final height was not affected.
		Ewing sarcoma	54		
Cool, et al. [16]	1998	Osteosarcoma	72	National Cancer for Health Statistic data	The final height was not affected.
This study	2020	Osteosacoma	24	Paley’s multiplier method	The final height was not affected.
				Japanese national growth curve data	

## Data Availability

All data are available from the corresponding author upon reasonable request.
